# Reciprocal Interactions between Medial Septum and Hippocampus in Theta Generation: Granger Causality Decomposition of Mixed Spike-Field Recordings

**DOI:** 10.3389/fnana.2017.00120

**Published:** 2017-12-12

**Authors:** Daesung Kang, Mingzhou Ding, Irina Topchiy, Bernat Kocsis

**Affiliations:** ^1^J. Crayton Pruitt Family Department of Biomedical Engineering, University of Florida, Gainesville, FL, United States; ^2^Department of Psychiatry, Beth Israel Deaconess Medical Center (BIDMC), Harvard Medical School, Harvard University, Boston, MA, United States

**Keywords:** theta oscillations, descending hippocampo-septal projections, sleep-wake states, REM sleep, active waking, slow wave sleep, medial septum neuron firing, freely moving rats

## Abstract

The medial septum (MS) plays an essential role in rhythmogenesis in the hippocampus (HIPP); theta-rhythmic bursts of MS neurons are believed to drive theta oscillations in rats’ HIPP. The MS theta pacemaker hypothesis has solid foundation but the MS-hippocampal interactions during different behavioral states are poorly understood. The MS and the HIPP have reciprocal connections and it is not clear in particular what role, if any, the strong HIPP to MS projection plays in theta generation. To study the functional interactions between MS and HIPP during different behavioral states, this study investigated the relationship between MS single-unit activity and HIPP field potential oscillations during theta states of active waking and REM sleep and non-theta states of slow wave sleep (SWS) and quiet waking (QW), i.e., sleep-wake states that comprise the full behavioral repertoire of undisturbed, freely moving rats. We used non-parametric Granger causality (GC) to decompose the MS-HIPP synchrony into its directional components, MS→HIPP and HIPP→MS, and to examine the causal interactions between them within the theta frequency band. We found a significant unidirectional MS→HIPP influence in non-theta states which switches to bidirectional theta drive during theta states with MS→HIPP and HIPP→MS GC being of equal magnitude. In non-theta states, unidirectional MS→HIPP influence was accompanied by significant MS-HIPP coherence, but no signs of theta oscillations in the HIPP. In theta states of active waking and REM sleep, sharp theta coherence and strong theta power in both structures was associated with a rise in HIPP→MS to the level of the MS→HIPP drive. Thus, striking differences between waking and REM sleep theta states and non-theta states of SWS and QW were primarily observed in activation of theta influence carried by the descending HIPP→MS pathway associated with more regular rhythmic bursts in the MS and sharper MS→HIPP GC spectra without a significant increase in MS→HIPP GC magnitude. The results of this study suggest an essential role of descending HIPP to MS projections in theta generation.

## Introduction

Patterns of local field potentials (LFPs) in rat hippocampus (HIPP) can be broadly classified as theta (4–10 Hz oscillations) and non-theta, which correspond to different behavioral states. Active exploration (AE) and rapid eye movement (REM) sleep are theta states, while quiet waking (QW) and slow-wave sleep (SWS) are non-theta states (Vanderwolf, 1969; Buzsáki, 2002).

The HIPP receives input from several cortical as well as subcortical areas, such as medial septum (MS). Specifically, the MS and the HIPP have reciprocal pathways (Raisman, 1966). In the MS, both GABAergic and cholinergic fibers project to HIPP (Frotscher and Léránth, 1985; Freund and Antal, 1988) whereas the HIPP projection to MS terminates on the GABAergic neurons in the MS (Tóth and Freund, 1992). The MS plays a critical role in regulating the electrical activity of the HIPP which provides information about the behavioral states of the animal (Khakpai et al., 2012). In particular, theta-rhythmic burst firing of MS neurons is thought to drive lasting HIPP theta oscillations in rats during waking motor activity and REM sleep (Petsche et al., 1962; Vertes and Kocsis, 1997; Buzsáki, 2002). Although the firing and other characteristics of theta bursting neurons in the MS have been studied in detail (Petsche et al., 1962; Ford et al., 1989; Sweeney et al., 1992; King et al., 1998; Dragoi et al., 1999), MS-hippocampal interactions during different behavioral states are poorly understood (Bland, 1986; Vertes and Kocsis, 1997).

It is not clear in particular what role, if any, the strong HIPP to MS projection plays in theta generation. That hippocampo-septal feedback is essential for the generation and maintenance of hippocampal theta oscillation has long been suggested in both experimental (Tóth et al., 1993) and modeling (Wang, 2002) studies. Moreover, the classical view of MS theta generation has been challenged by demonstrating *in vitro* the capability of HIPP networks to generate theta independently without the MS (Manseau et al., 2008), suggesting a hypothesis of hippocampal lead over the MS in the regulation of theta rhythm. A recent study provided experimental evidence that HCN (hyperpolarization-activated cyclic nucleotide-gated nonselective cation channel) immunonegative neurons in the MS form a septal follower group, which receive rhythmic inputs from hippocampal and/or from GABAergic MS neurons in urethane-anesthetized rats (Hangya et al., 2009).

In freely moving rats, we have found further support for the critical role of a descending HIPP to MS rhythmic drive in theta generation by contrasting SWS, a non-theta state, against microarousals, short theta events frequently interrupting SWS (Kang et al., 2015). It’s been known that in non-theta states a group of MS neurons (8% in SWS of unanesthetized rats and 20% in urethane anesthesia, Sweeney et al., 1992) exhibits rhythmic burst firing in the theta range which does not lead to HIPP theta. Using Granger causality (GC) measure, we demonstrated that these neurons exhibit significant MS→HIPP GC in SWS, but when theta appears during microarousals, the unidirectional MS→HIPP drive switches to a bidirectional MS-HIPP relationship, in which MS→HIPP remains unchanged but a significant HIPP→MS emerges and rises to the same level as MS→HIPP (Kang et al., 2015).

To further study the functional interactions between MS and HIPP during different behavioral states, we now extended the Kang et al. (2015) study to the investigation of the relationship between single-unit activity in MS and LFP oscillations in HIPP during lasting theta states (AE, REM) and non-theta states (SWS, QW), i.e., sleep-wake states that comprise the full behavioral repertoire of undisturbed, freely moving rats. Using the same experimental and analysis techniques in this study we were able to generalize the conclusions drawn in the previous study in a specific case (Kang et al., 2015); by simultaneously recording MS unit activity with HIPP LFP during theta and non-theta states in freely behaving rats, we analyzed power, coherence and GC and found that theta rhythm in HIPP is accompanied in all behavioral theta states by a strong descending HIPP to MS drive, whose magnitude equals or even exceeds the theta drive from the MS to HIPP. These results were obtained by using a recently introduced non-parametric GC method developed for mixed spike-field recordings, where one signal is a continuous-valued signal and the other a point process. In this method, GC, along with power and coherence, is estimated directly from Fourier transforms of data without the need for autoregressive (AR) models. For validation, the proposed GC method was tested on simulated data of two-node and three-node network models, and shown to recover the known connectivity patterns (see Supplementary Data).

## Materials and Methods

### Experimental Procedures

Male Sprague-Dawley rats were treated in accordance with National Institutes of Health guidelines. All experimental procedures were approved by Institutional Animal Care and Use Committee of Beth Israel Deaconess Medical Center. The experimental procedures were described earlier in detail (Kang et al., 2015). Briefly, the rats were deeply anesthetized for implantation of stainless steel wires for recording HIPP LFP, stainless steel screws for reference, ground and cortical EEG recording, and multithreaded wires for recording neck muscle activity (EMG). For MS unit recording, three tetrodes were mounted on individually movable microdrives and lead into a guide tube placed above the MS (AP +0.5 mm, Lat 0.0 mm, DV −3.0 mm). Electrophysiological recordings started after a 7–10 day recovery period. Daily recording sessions lasted 2–6 h during daylight period, in a 26 × 17 × 17 cm recording box. After stable LFP and EMG recordings were attained, the tetrodes were moved slowly into the MS until discriminable unit activity were found; tracks which had at least one theta-rhythmic single unit were considered for further analysis. The electrical signals were amplified, filtered (LFP: 0.1–100 Hz, EMG: 0.1–3 kHz, units: 600–3 kHz) and sampled (16-bit, 10 kHz; Neuralynx Inc.). MS single neurons were identified and extracted off-line based on their amplitude and wave-shape using principal component and K-means clustering algorithms (Spike2, Cambridge Electronic Devices, UK). Units showing a refractory period of 2 ms or higher were considered as single units. All neurons encountered in these recording sites were then included in the analysis independent of their firing properties. The spike trains of identified MS units along with HIPP LFP signals in each behavioral state were transferred to MATLAB for analysis.

The MS electrode location was marked at the end of the experiment by direct current to generate lesions at different dorsoventral locations which together with the damage caused by the guide cannula above the MS served for verification of the microelectrode placement in the MS. The dorsoventral location of individual neurons along this axis was estimated by the number of turns of the Microdrive. The study used the same set of rats described previously (Kang et al., 2015) but the criteria for neuron selection included lasting theta or non-theta states. Theta rhythmic cells were encountered along the MS midline in three of four rats; in one rat in which electrode tracks were found more lateral no theta cells were found and thus this animal was excluded from the analysis. A total of 70 cells were recorded in QW, 79 cells in SWS, 80 cells in AE and 57 cells in REM sleep.

The rats’ behavior was undisturbed during recording sessions. Behavioral states, i.e., sleep-wake states, such as AE, QW, SWS and REM sleep, were identified according to standard polysomnographic evaluation criteria (Vanderwolf, 1969; Vertes and Kocsis, 1997; Ly et al., 2013) based on visual sleep scoring aided by auxiliary signals representing running averages of EMG total power, EEG power in the delta range (1–4 Hz) over the frontal cortex, and in the theta range (5–10 Hz) over the parietal cortex and HIPP, as well as the delta-to-theta ratio. AE was characterized by concurrent high theta and high EMG activity, QW by lower EMG and mixed and variable EEG oscillations, SWS by low EMG activity, high delta, and low theta power, and REM sleep by high theta and minimal EMG.

### Data Analysis

HIPP LFP and MS spike trains were subjected to spectral analysis. In addition to power spectra from each structure, the MS-HIPP coherence spectra were estimated to represent the interaction between the two structures (Jenkins and Watts, 2000). The MS-HIPP interaction was further decomposed into their directional components, MS→HIPP and HIPP→MS, using a recently proposed non-parametric GC algorithm designed for point processes as well as continuous-valued recordings (Dhamala et al., 2008a,b; Nedungadi et al., 2009). To the best of our knowledge, application of GC to mixed time series has not been addressed before the technical development we published in Kang et al. (2015), so we will explain again the method below and point out the relevant references. In the Supplementary Data, we apply the method to numerical models to test and illustrate the application of the non-parametric GC to mixed recordings of continuous-valued time series and point processes.

Typically, GC for continuous-valued signals is estimated by fitting parametric AR models to experimental data (Ding et al., 2006). For discrete time series such as spike train data, however, it is difficult to apply this approach since AR model of spike train data is not readily obtainable. To resolve this issue, several approaches have been attempted (Sameshima and Baccalá, 1999; Fanselow et al., 2001; Kamiński et al., 2001; Zhu et al., 2003; Nedungadi et al., 2009; Kim et al., 2011), including converting the spike train data into continuous-valued time series by using a low pass filter (Kamiński et al., 2001; Zhu et al., 2003) or a smoothing kernel (Sameshima and Baccalá, 1999; Fanselow et al., 2001). While these approaches have been applied to both simulated and experimental data with generally acceptable results, it is cautioned that the smoothing operation violates the point process character of spike trains. Furthermore, the approaches are highly kernel dependent and may introduce unwanted distortions (Truccolo et al., 2005). More recently, to tackle discrete time series in GC application, a non-parametric GC method (Nedungadi et al., 2009) and likelihood based GC (Kim et al., 2011) have been proposed, which yielded promising results when applied to both simulated and experimental data. Although GC between continuous-valued time series or GC between point process data are mathematically well-defined, GC between LFP and spike trains, referred to as mixed recordings or mixed signals, is not well-understood. In order to obtain directionality between mixed signals, we extended the non-parametric GC to the mixed time series of LFP and spike train data by combining spectral matrix factorization of mixed time series with Geweke’s spectral formulation of GC.

The procedure of data analysis is as follows. The continuous recordings were divided into 2 s non-overlapping epochs which were treated as realizations of an underlying stochastic process. Over 99% of the LFP epochs met the stationarity requirement according to the KSPP test (Kwiatkowski et al., 1992). Each epoch was divided into 1 ms bins so that no more than one spike can be found in any bin. HIPP LFP and MS spike train were subject to separate Fourier transforms; averaging auto-spectra and cross-spectra across all the recording epochs within a behavioral state yielded the spectral density matrix, from which power and coherence can be derived. The spectral density matrix was further factorized and combined with Geweke’s spectral GC formalism to yield MS→HIPP GC and HIPP→MS GC in the spectral domain (Ding et al., 2006; Geweke, 1982). A random permutation procedure was used to generate the significance thresholds for coherence and GC. Specifically, for each neuron, the epoch labels for LFP and the epoch labels for spike train were permuted randomly 1000 times. Coherence and GC were computed for each of the 1000 permuted datasets. Null hypothesis distributions were constructed based on these synthetic coherence and GC values. Thresholds corresponding to *p* = 0.01 were determined and neurons whose coherence or GC was above their respective thresholds were considered statistically significant.

For a given metric (e.g., firing rate, peak coherence frequency, et cetera), statistical comparisons was carried out using one way ANOVA to test whether the four states showed significant differences, which was followed by *ad hoc* multiple comparison test with 95% confidence interval to reveal pairwise differences between behavioral states.

## Results

Electrophysiological recordings were conducted in different sleep-wake and behavioral states in which hippocampal activity can be broadly classified into two distinct LFP patterns, theta and non-theta. Theta states included two behavioral states, AE and REM sleep, while non-theta states included QW and SWS. In AE, animals were engaged in exploratory behaviors (locomotion, sniffing and whisking), which were characterized by voluntary motor activity. During REM sleep, animals were immobile and atonic except for intermittent whisker and ear twitches. Both AE and REM sleep exhibited low-amplitude LFPs and high theta (5–9 Hz) and gamma (30 –55 Hz) power spectrum density and are thus considered theta states. In QW, animals are immobile (standing or sitting quietly) or engaged in automatic stereotyped behaviors (eating, drinking and grooming); these behaviors were characterized by low-amplitude LFPs and absence of theta, and is thus considered a non-theta state. During SWS, animals were lying immobile with eyes closed and with slow regular respiratory movements. The LFPs exhibited high-amplitude slow waves mainly in delta band (1–4 Hz); SWS is thus also a non-theta state (Vanderwolf, 1969; Buzsáki, 2002).

Conventionally, state-dependent hippocampal activity is thought to be under control by ascending input from the brainstem arousal system, conveyed by the MS rhythmically firing in synchrony with hippocampal LFP. To what extent HIPP activity influences the dynamics of the MS-HIPP circuit remains unclear despite the fact that it has been known since the 1960s that the MS and the HIPP have reciprocal pathways (Raisman, 1966).

**Figure 1:**
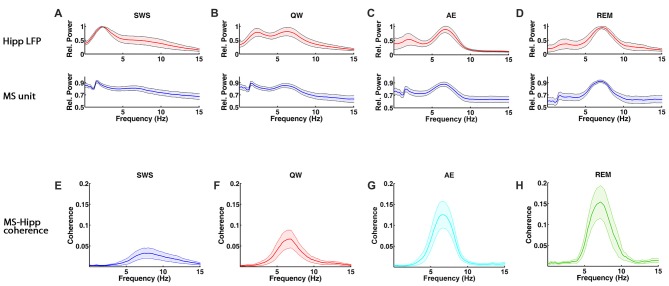
Spectral characteristics of medial septum (MS) neuron and hippocampal local field potential (LFP) activity and MS-hippocampal coherence during theta and non-theta states, slow wave sleep (SWS), quiet waking (QW), active exploration (AE) and rapid eye movement (REM) sleep. **(A–D)** Group averaged relative power spectra of hippocampal LFP (top panels) and MS activity (bottom). **(E–H)** Group averaged coherence between hippocampal LFP and MS activity.

**Figure 2:**
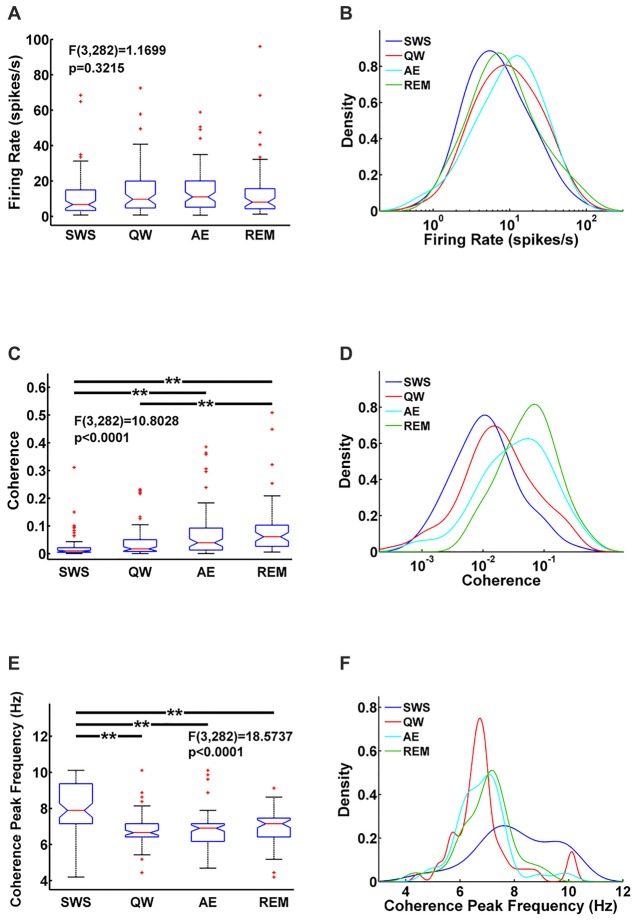
Comparison of MS units firing rate **(A)** MS-hippocampus (HIPP) coherence **(C)** and coherence peak frequency **(E)** for each state and their corresponding densities **(B,D,F)** in each state. Significance indicators “**” represents *p* < 0.005.

MS unit activity and HIPP LFP were recorded simultaneously to address this question. In MS, a total of 70 cells was recorded in QW, 79 cells in SWS, 80 cells in AE, and 57 cells in REM sleep. All cells were included in the analysis independent of their firing properties. Both MS unit activity and HIPP LFP power spectra showed prominent theta peaks (range 4–10 Hz) in AE and REM sleep, whereas delta activity (ranges 1–3 Hz) dominated HIPP LFP during SWS (Bland, 1986; Figures 1A–D). The QW stage showed both delta and theta peaks in power spectra of MS unit activity and HIPP LFP. The firing rate of the MS units was in the range as reported in earlier studies (Ford et al., 1989; Sweeney et al., 1992; King et al., 1998; Dragoi et al., 1999) and was not significantly different (*F*_(3,282)_ = 1.1699, *p* = 0.3215) among SWS (10.73 ± 2.70 spikes/s), QW (14.13 ± 3.25 spikes/s), AE (14.13 ± 2.71 spikes/s), and REM sleep (14.41 ± 4.76 spikes/s; Figure 2A). The overlapping MS firing rate distributions in Figure 2B provided further evidence that unit firing rates in MS were not significantly different across the four behavioral states.

Functional MS-HIPP interactions were first investigated using spectral coherence (Figures 1E–H). Mean theta range coherences were significantly different (*F*_(3,282)_ = 10.8028, *p* < 0.0001); they were larger in theta states (Figures 1G,H; AE: 0.0696 ± 0.0188, and REM sleep: 0.0892 ± 0.0258) than in non-theta states (Figures 1E,F; SWS: 0.0238 ± 0.0094, QW: 0.0405 ± 0.0138). Comparison of coherence for different state pairs showed that SWS vs. AE (*p* = 0.0005), SWS vs. REM (*p* < 0.0001), and QW vs. REM (*p* = 0.0012) were significantly different while the rest of state pairs (SWS vs. QW: *p* = 0.5092, QW vs. AE: *p* = 0.0735, AE vs. REM: *p* = 0.4122) were not (Figure 2C).

In contrast to MS unit firing rate, the coherence spectra across different units indicated unequal distributions in different states; during theta states, coherence was biased to the right whereas during non-theta states, coherence was biased to the left (i.e., to higher and lower coherences, respectively; Figure 2D). The frequencies of maximal coherences also showed state-dependent bias (Figure 2F); peak coherence frequency during SWS had two peaks around 8 Hz and 10 Hz and peak coherence frequencies during other states resided between 6 Hz and 7 Hz. The mean coherence peak frequencies (SWS: 8.0256 ± 0.3251, QW: 6.8592 ± 0.2593, AE: 6.8433 ± 0.2118, and REM: 6.9656 ± 0.2530) were significantly different (*F*_(3,282)_ = 18.5737, *p* < 0.0001). Further analysis showed that the mean coherence peak frequency during SWS was significantly different from the other states: SWS vs. QW (*p* < 0.0001), SWS vs. AE (*p* < 0.0001), and SWS vs. REM (*p* < 0.0001); no difference was found in the following comparisons: QW vs. AE (*p* = 0.9998), QW vs. REM (*p* = 0.9560), and AE vs. REM (*p* = 0.9300; Figure 2E).

**Figure 3:**
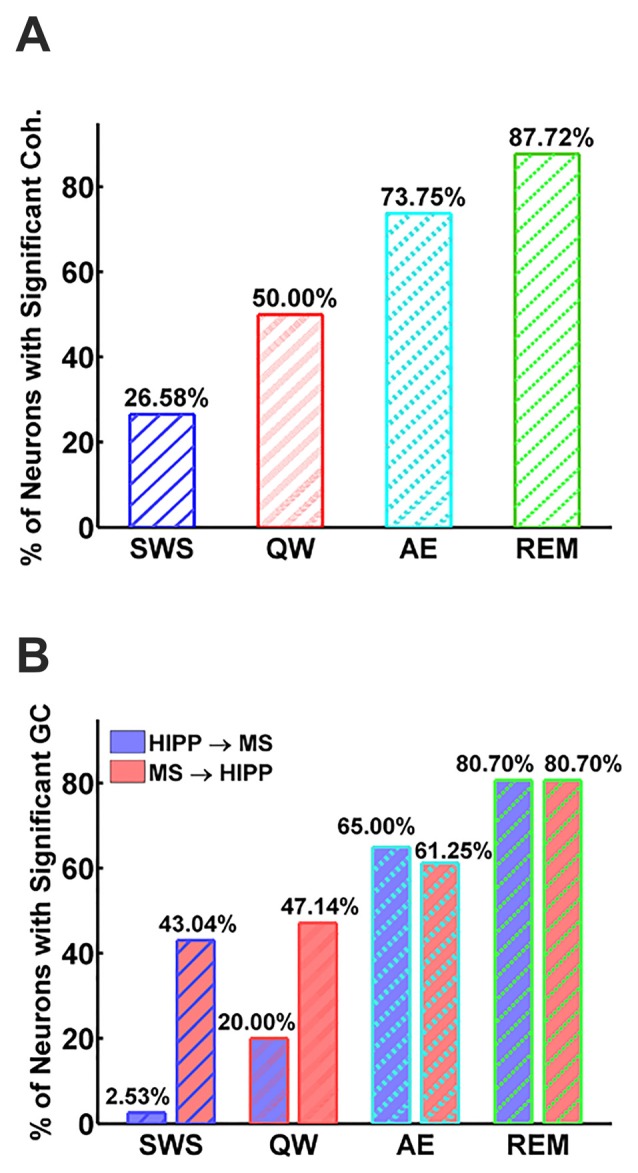
Percentage of neurons with significant MS-Hipp coherence **(A)** and significant MS→HIPP or HIPP→MS Granger causality (GC; **B**).

**Figure 4:**
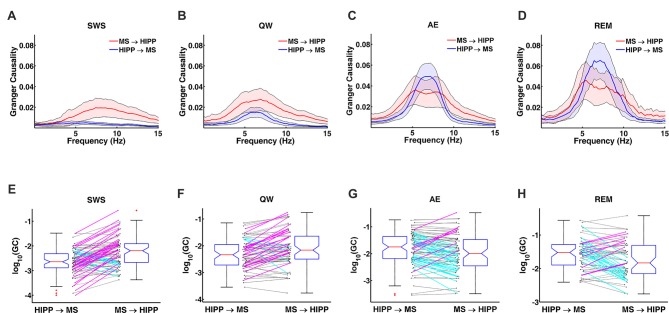
Changes in GC during non-theta (SWS, QW) and theta (AE, and REM sleep) states. **(A–D)** Group averaged GC between hippocampal LFP and MS activity in MS→HIPP (red) and HIPP→MS directions (blue). **(E–H)** GC of individual neurons in the two directions for each state.

Next, MS-HIPP interactions were further investigated with GC. Compared to coherence, which is non-directional, GC offers the advantage of decomposing these interactions into ascending (MS→HIPP) and descending directions (HIPP→MS). The number of MS neurons with significant coherence with HIPP LFP showed an increasing trend from SWS (26.58%) to QW (50.00%) during non-theta states; a further increase was seen going from non-theta to theta states (AE: 73.75%, REM sleep: 87.72%; Figure 3A). The number of neurons with significant theta range GC showed a more complex relationship, in which the proportion in GC in the two directions were clearly distinguishing theta and non-theta states (Figure 3B). Average GC spectra also show a state-dependent bias from unidirectional (in SWS and GC) to bidirectional MS-HIPP interactions in theta states of AE and REM sleep (Figures 4A–D). The striking difference is further demonstrated on the single unit level in Figures 4E–H, in which magenta lines, marking greater MS→HIPP GC than HIPP→MS GC, dominate in SWS, and whereas blue lines, marking lower MS→HIPP GC than HIPP→MS GC, dominate in AE and REM sleep. The differences were significant in statistical analysis, in which bootstrapping with 1000 random sampling with replacement were conducted to construct null hypothesis for each neuron with 95% confidence interval.

**Figure 5:**
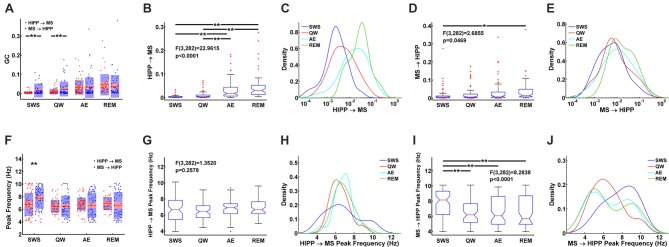
Statistical comparison of HIPP→MS and MS→HIPP GC. **(A)** Average and distribution of HIPP→MS and MS→HIPP GC for each state. **(B–E)** GC based on GC direction during SWS, QW, AE, and REM sleep. Comparison of HIPP→MS **(B)** and its density **(C)** vs. MS→HIPP **(D)** and its density **(E)**. **(F)** Peak frequencies of HIPP→MS and MS→HIPP GC for each state. **(G–J)** Comparison of peak frequency **(G)** and the density of HIPP→MS **(H)** vs. peak frequency of MS→HIPP (average **(I)** and density **(J)**). Significance indicators “*” and “**” represents *p* < 0.05 and *p* < 0.005, respectively.

During SWS, the percentage of neurons with significant theta range GC was 2.53% for HIPP→MS and 43.04% for MS→HIPP. Thus, GC indicated a nearly unidirectional MS→HIPP drive in which MS neuronal activity affected HIPP LFP at frequencies within the theta range (group average of GC = 0.016), but the firing activity of these neurons was much less affected by HIPP activity (GC = 0.0042; Figure 3B). MS→HIPP GC was significantly greater than HIPP→MS GC (paired *t*-test, *t*_(78)_ = −3.3202, *p* = 0.0014; Figure 5A). In QW state, the proportion of neurons with significant HIPP→MS GC increased to 20.00%, but the proportion of neurons with significant MS→HIPP GC remained essentially the same (47.14%). Although HIPP→MS GC increased compared with SWS, the GC between HIPP→MS (group average GC = 0.0103) and MS→HIPP (GC = 0.0231) was significantly different in paired *t*-test (*t*_(69)_ = −3.8291, *p* = 0.0003; Figure 5A). These results from non-theta states indicated that causal interaction from MS to HIPP is significantly greater than that in the opposite direction.

During theta states, the interaction became bidirectional, with the advent of a strong descending HIPP→MS GC component (group average: 0.0317 for AE and 0.0458 for REM sleep) and consistent ascending MS→HIPP GC component (group average: 0.0309 for AE and 0.0376 for REM sleep). The percentage of neurons with significant GC increased for both HIPP→MS (65.00% in AE and 80.70% in REM sleep) and MS→HIPP (61.25% and 80.70%) directions (Figure 3B). GC from HIPP to MS (Figure 5B) was significantly different (*F*_(3,282)_ = 22.9615, *p* < 0.0001) among SWS (0.0042 ± 0.0013), QW (0.0103 ± 0.0034), AE (0.0317 ± 0.0084), and REM sleep (0.0458 ± 0.0144). Specifically, non-theta states had significantly different GC means compared with theta states: SWS vs. AE (*p* < 0.0001), SWS vs. REM (*p* < 0.0001), QW vs. AE (*p* < 0.0004), and QW vs. REM (*p* < 0.0001). The mean differences within non-theta states (SWS vs. QW: *p* = 0.6752) and theta states (AE vs. REM: *p* = 0.0646) were not significantly different. The densities of HIPP→MS were clearly separated to the left during non-theta states and to the right during theta states as shown in Figure 5C. The MS→HIPP GC among four different states (SWS: 0.0162 ± 0.0079, QW: 0.0231 ± 0.0089, AE: 0.0309 ± 0.0118, and REM: 0.0376 ± 0.0153) was significantly different (*F*_(3,282)_ = 2.6855, *p* = 0.0469; Figures 5D,E). In pairwise comparisons, SWS showed significantly different means from REM sleep (*p* = 0.0434) while other pairs of states were not significantly different (SWS vs. QW: *p* = 0.8070, SWS vs. AE: *p* = 0.1959, QW vs. AE: *p* = 0.7380, QW vs. REM: *p* = 0.3083 and AE vs. REM: *p* = 0.8462). Moreover, in theta states, HIPP→MS GC and MS→HIPP GC no longer differed in paired *t*-test (AE: *t*_(79)_ = 0.1807, *p* = 0.8571 and REM: *t*_(56)_ = 1.4285, *p* = 0.1587).

Furthermore, the peak frequencies of HIPP→MS GC spectra (Figure 5F) (SWS: 6.7770 ± 0.4042, QW: 6.4752 ± 0.2346, AE: 6.8218 ± 0.2276, and REM: 6.8920 ± 0.2709) were not significantly different among the four behavioral states (*F*_(3,282)_ = 1.3520, *p* = 0.2578; Figures 5G,H). Likewise, there were no significant differences between pairs of states (SWS vs. QW: *p* = 0.4911, SWS vs. AE: *p* = 0.9964, SWS vs. REM: *p* = 0.9571, QW vs. AE: *p* = 0.3631, QW vs. REM: *p* = 0.2755, and AE vs. REM: *p* = 0.9895). In contrast, the peak frequency of MS→HIPP GC spectra (SWS: 7.7915 ± 0.3693, QW: 6.4858 ± 0.3945, AE: 6.6121 ± 0.4309, and REM: 6.6324 ± 0.5487) showed significant difference (*F*_(3,282)_ = 8.2838, *p* < 0.0001; Figures 5I,J). SWS had significantly different GC peak frequency from the other states: SWS vs. QW (*p* = 0.0001), SWS vs. AE (*p* = 0.0004), and SWS vs. REM (*p* = 0.0018), whereas QW vs. AE (*p* = 0.9757), QW vs. REM (*p* = 0.9709), and AE vs. REM (*p* = 0.9999) were not significantly different. The peak frequency of HIPP→MS GC was significantly different compared with MS→HIPP (paired *t*-test *t*_(78)_ = −4.2617, *p* < 0.0001) during SWS but not in any other state (QW (*t*_(69)_ = −0.0424, *p* = 0.9663, AE (*t*_(79)_ = 0.9410, *p* = 0.3495, and REM sleep; *t*_(56)_ = 0.8628, *p* = 0.3919 when paired *t*-test was performed).

## Discussion

The results of this study suggest an essential role of descending HIPP to MS projections in theta generation during natural theta states in both sleep and wake, supporting and extending the model proposed previously based on the analysis of microarousals, which is a special theta state (Kang et al., 2015). In previous investigations, the anatomy of the HIPP to MS projection has been described in detail (Tóth et al., 1993) but its function remained unclear. In the current study, we used non-parametric GC to decompose the MS-HIPP synchrony into its directional components and to examine the causal interactions between them within the theta frequency band during theta (AE, REM) and non-theta (SWS, QW) states. The main finding was that there is a significant unidirectional MS→HIPP influence in non-theta states which switches to bidirectional theta drive during lasting theta states of AE and REM sleep, with MS→HIPP and HIPP→MS GC being of equal magnitude. Thus, the results of this study extend our previous findings from specific SWS-microarousal alterations to all major non-theta to theta transitions. In SWS, we found unidirectional MS→HIPP influence accompanied by significant MS-HIPP coherence, but no signs of theta oscillations in the HIPP. During QW, HIPP→MS GC slightly increased compared with SWS; however, the increase was not significant. In the theta states of AE and REM sleep, sharp theta coherence and strong theta power in both structures was associated with a rise in HIPP→MS to the level of the MS→HIPP drive. Thus, striking differences between theta states of AE and REM sleep and non-theta states of SWS and QW were primarily observed in activation of theta influence carried by the descending HIPP→MS pathway, which was associated with: (1) more regular rhythmic bursts in the MS; (2) increased synchronization of MS→HIPP and HIPP→MS as evidenced by peak frequencies being at ~6 Hz in both GC spectra; and (3) sharper MS→HIPP GC spectra without a significant increase in MS→HIPP GC magnitude. Analytically, these findings were made possible by extending the non-parametric GC method to mixed-signal recordings, which in the present experiment contain one continuous-valued signal (HIPP LFP) and one point process signal (MS spikes). This analytical approach, first proposed in Kang et al. (2015), was further validated by analyzing mixed signals generated by simulated network models, where we showed that the patterns of network connectivity were correctly recovered (Supplementary Figures S1, S2).

It’s been known for over 50 years, that during theta states, including AE and REM sleep, when HIPP LFP is dominated by regular 4–10 Hz oscillations (Buzsáki, 2002), MS neurons fire rhythmic bursts in synchrony with HIPP LFP (Petsche et al., 1962). The long-standing MS pacemaker theory, in which rhythmic MS cells drive HIPP theta, also received further support from numerous lesion studies and neuropharmacological studies (Lawson and Bland, 1993). Systematic investigations of MS theta neuron firing in freely moving rats, however, were sporadic (Ford et al., 1989; King et al., 1998; Dragoi et al., 1999; Jinno et al., 2007; Vandecasteele et al., 2014). MS unit autocorrelograms showing weaker or transient theta frequently appeared in the literature (Macadar et al., 1970; Ranck, 1976; Alonso et al., 1987; Dutar et al., 1995; Apartis et al., 1998). These data have been interpreted in the framework of the MS theta pacemaker hypothesis contending that the major difference between theta and non-theta states were in the number of MS theta bursting cells; in theta states, larger numbers of theta bursting cells provide a strong theta rhythmic input to HIPP to drive HIPP theta LFP response, whereas in non-theta states, the weak theta rhythmic drive from smaller numbers of MS theta bursting cell is insufficient to elicit theta field oscillations in HIPP. This concept has solid a physiological foundation; the number of theta bursting MS neurons in unanesthetized, head-restrained rats did indeed show a strong increase from non-theta to theta states (8% in SWS, 64% in active waking, 94% in REM sleep; Sweeney et al., 1992) which was also replicated in rats anesthetized with urethane (20% and 59% in non-theta and theta states, respectively). Our study provides further evidence for the undisputable role of MS neural activity in HIPP theta generation. The number of MS neurons firing rhythmic bursts in synchrony with HIPP theta LFP showed strong increase; neurons with significant coherence increased from 26% in SWS to 88% in REM sleep, and the percentage of neurons with significant MS→HIPP GC increased from 43% to 81% from SWS to REM sleep, although the differences were not significant in other pairwise comparisons of theta (61% in AE) and non-theta states (47% in QW).

The overwhelming majority of prior investigations of the relationship between MS and HIPP focused on the role of MS input in controlling hippocampal activity and did not account for the possible role of the descending HIPP to MS projection (Tóth et al., 1993). The present study is among the firsts to quantify the effects of both limbs of this reciprocal projection, by estimating the effects of the MS→HIPP and HIPP→MS drives in different theta and non-theta states and identifying the drastic increase in HIPP→MS as the most significant change in all theta states compared with non-theta states. During all theta states, both in wake and sleep (AE and REM sleep), GC values were approximately equal in the two directions, namely, MS→HIPP ≈ HIPP→MS, whereas the relationship was unidirectional pointing from MS to HIPP in non-theta states both in waking (QW) and sleeping (SWS) animals, namely, MS→HIPP >> HIPP→MS. AE and REM sleep was characterized with increased MS and HIPP activity (Buzsáki, 2002) and somewhat higher MS→HIPP (only significant in SWS vs. REM sleep comparison) which, however, did not change this relationship. This is also supported by our previous observation comparing GC in SWS and short (<10 s) microarousals (Kang et al., 2015) where the switch from unidirectional to bi-directional patterns was also accompanied by enhanced HIPP theta oscillations when the MS→HIPP remained at the same level as in SWS. Furthermore, the significant MS→HIPP GC during SWS did not lead to manifest hippocampal theta, indicating that the “weak, considered subthreshold, MS to HIPP input during SWS is not sufficient to explain the lack of theta LFP during SWS” (Kang et al., 2015). The findings of this study turns the emphasis to the critical role of HIPP network responsiveness to MS input in the non-theta to theta switch during AE and REM sleep which may then induce intrinsic theta in HIPP networks (Manseau et al., 2008). GC analysis also indicates that activating the HIPP→MS theta drive acts by synchronizing the MS pacemaker rather than by increasing MS→HIPP drive over a threshold to drive theta in the HIPP.

Computational modeling has long implicated reciprocal interactions between MS and HIPP as an important determinant underlying HIPP network activities. Our findings are in agreement with predictions by a model proposed by Wang (2002), in which the emergence of robust theta synchrony requires the addition of a second GABAergic population projecting to the pacemaker, even if membrane properties enable this latter to generate theta rhythmic discharge on its own (Serafin et al., 1996). As indicated by the massive HIPP→MS GC emerging in theta states, the descending HIPP to MS GABAergic input (Tóth and Freund, 1992; Tóth et al., 1993) may fulfil the role of this second population. The effect of descending theta drive on single unit burst firing was also shown in response to sensory stimulation (tail pinch) in subsets of MS neurons (Hangya et al., 2009) and posterior hypothalamus (Kocsis, 2006; Kocsis and Kaminski, 2006; Ruan et al., 2017). In a more recent model (Hangya et al., 2009), based on theta elicited by brief (10 s) sensory stimulation under urethane anesthesia and presented in the framework of the MS theta pacemaker hypothesis, the switch to the “formation of population level theta rhythm” included a crucial mechanism by which synchrony is rapidly enhanced in both structures by the “reciprocal septo-hippocampal dialog”.

Between theta and non-theta states, MS→HIPP GC remains approximately the same, yet the HIPP responses to the MS input differed drastically. The neuronal mechanisms and the role of HIPP gating in the non-theta to theta switch remains to be further investigated. State-related control of HIPP activity parallels ascending control of the cortex, exerted by the brainstem, and using region-specific slow oscillations which involve subcortical systems, such as the septo-hippocampal system in the HIPP and thalamo-cortical networks in the cortex, for their generation (Kim et al., 2017). Thus, emergence of HIPP theta, strongly related to behavioral and sleep-wake states, controlled by brainstem arousal mechanisms (Buzsáki, 2002; Saper et al., 2005) including several subcortical, aminergic and cholinergic, neurotransmitter systems (Pace-Schott and Hobson, 2002). These systems, in addition to their effect on septo-hippocampal control (Kocsis and Vertes, 1994, 1996), also act locally within the HIPP and may modulate oscillatory activity of neural networks. For example, histaminergic and norepinephrinergic pathways, originating in the brainstem, densely innervate the HIPP and were shown to modify theta through local HIPP receptors (Hajós et al., 2003; Masuoka and Kamei, 2007). Histamine and norepinephrine were also shown to enhance HIPP theta *in vivo*, using systemic drug administration (Kocsis et al., 2007; Hajós et al., 2008; Ly et al., 2013). The critical role of the cholinergic system, as an important component of the MS to HIPP projection, has also been known for decades; lesioning MS cholinergic neurons selectively, without affecting the MS GABAergic output, dramatically reduces theta amplitude (Lee et al., 1994; Apartis et al., 1998), although has no effect on theta frequency. The activation dynamics of cholinergic receptors on HIPP neurons are slow to directly drive 4–10 Hz oscillations but cholinergic tone ascending from the MS can change the receptiveness of HIPP networks to induce HIPP theta and activate the HIPP→MS theta drive. A descending cholinergic output also originating in the MS has also been shown recently to exert a theta promoting effect in the supramammillary nucleus (Arrifin et al., 2017), another subcortical structure expressing theta rhythmic neuronal firing (Kocsis and Vertes, 1997) and an essential component theta generation (Kocsis and Vertes, 1994).

Our findings are also in agreement with recent optogenetic investigations of GABAergic and cholinergic neurons of the MS to HIPP pathway (Vandecasteele et al., 2014; Dannenberg et al., 2015). According to firing rate and other characteristics (Matthews and Lee, 1991; King et al., 1998), theta rhythmic MS cells in our study were most likely GABAergic whereas cholinergic neurons activated in AE and REM sleep may be responsible for modification of network properties in both HIPP and MS. The MS GABAergic population, when selectively activated (Dannenberg et al., 2015), induced oscillations at the higher end of theta frequencies (~10–15 Hz) but when theta at lower frequencies (~4 Hz) were induced by parallel cholinergic input they fired in synchrony with HIPP theta rhythm. This shift, recorded under urethane anesthesia in mice, might be analogous to the shift in the peak frequency of MS→HIPP GC from high theta in SWS to lower theta (6–7 Hz) band in theta states of AE and REM sleep, observed in our study in behaving rats, i.e., without anesthesia. Selective optogenetic activation of MS cholinergic neurons showed remarkable differences between anesthetized and un-anesthetized mice (Vandecasteele et al., 2014). It suppressed HIPP LFP activity at peri-theta frequencies, i.e., below (0.5–4 Hz) and above (10–25 Hz) theta in both preparations, sharpening the spectrum at theta, i.e., increasing theta/slow oscillation ratio. However, enhancement of theta power was only induced under anesthesia whereas in behaving animals, it decreased or remained unchanged. MS cholinergic-induced HIPP theta sharpening was most effective in SWS. Our data suggest, that such selective cholinergic activation might have primarily affected HIPP responsiveness, activated the descending HIPP to MS pathway and synchronized the firing in the MS pacemaker. The small subset of MS neurons firing rhythmic theta bursts in SWS (Sweeney et al., 1992) and showing significant coherence with HIPP LFP (26% in our study), was probably insufficient to increase theta amplitude. In AE and REM sleep the MS cholinergic pathway is already active rendering attempts of further activation of MS cholinergic neurons ineffective. Future studies of co-application of GC and optogenetic stimulation may shed further light on this issue and test these hypotheses.

## Author Contributions

DK, MD and BK analyzed the data, interpreted the results and wrote the manuscript. IT and BK performed the experiments.

## Conflict of Interest Statement

The authors declare that the research was conducted in the absence of any commercial or financial relationships that could be construed as a potential conflict of interest.
