# Supplementary material for: Reciprocal Interactions between Medial Septum and Hippocampus in Theta Generation: Granger Causality Decomposition of Mixed Spike-Field Recordings
Source: Front Neuroanat. 2017 Dec 12;11:120. doi: 10.3389/fnana.2017.00120 (PMC5733038; doi:10.3389/fnana.2017.00120)
Supplement: Supplementary file 1 [file Data_Sheet_1.pdf]

Supplemental material

1. Granger causality via factorization of spectral density matrix

For two continuous-valued time series, in the conventional parametric approach, an AR model is fit to the data. From the model one computes the spectral density matrix according to

$$S(f) = H(f)\Sigma H^{*}(f), \quad (1)$$

where the asterisk denotes matrix transposition and complex conjugation, $H(f) = \left(\sum_{k=0}^{m} A_k e^{-2\pi ikf}\right)^{-1}$ is the transfer function which depends on the coefficients of the AR model, and Σ is the covariance matrix of the error terms in the AR model (Ding et al. 2000, 2006). The power spectra and coherence can be obtained from $S(f)$ whereas Granger causality calculation required information from all three entities in Equation (1). For mixed signal recordings where one or more time series are point processes (spike trains), the AR model approach is not viable, and therefore a nonparametric approach is preferred. In the nonparametric approach, the spectral density matrix $S(f)$ is estimated from data using Fourier transform, a common practice, and from $S(f)$ power spectra and coherence are readily computed. To compute GC, however, we require the other two entities in Equation (1). These can be accomplished by applying the technique of spectral density matrix factorization (Wilson 1972), which decomposes $S(f)$ into a unique corresponding transfer function $H(f)$ and the noise covariance matrix Σ (Dhamala et al. 2008a, b), as in Equation (1). From these three entities Granger causality between continuous-valued signals (e.g. LFP) and point process (e.g., spike trains) can then be assessed.

2. Summary of the algorithm

A step by step algorithm for computing non-parametric GC between LFP and spike trains is as follows.

Step 1. The spike trains generated by a neuron are taken as one realization of stochastic point process denoted by $N_1(t)$. The spike trains are binned where the bin width was pre-chosen (in this dissertation, the bin width is 1 ms). When we define smaller bin width, many of the bins will contain no spikes leading to poor estimation of the spectral density matrix.

Step 2. The Fourier transform of $x(t)$ and $N_1(t)$ are estimated using equation (2-2) and (2-3), respectively.

Step 3. The spectral density matrix $S(f)$ for the time series $x(t)$ and stochastic process $N_1(t)$ is obtained by using equation (2-7).

Step 4. The spectral density submatrix is factorized using the spectral factorization algorithm (Wilson 1972; Dhamala et al. 2008a, b), thus giving the decomposition in equation (2-8).

Step 5. GC from LFP $x(t)$ to spike trains $N_1(t)$ is evaluated as a function of frequency by substituting the transfer function $H(f)$ and the noise covariance matrix Σ in equation (2-10). This function can be examined for frequency characteristics of causal influences or summed over all frequencies to obtain a single time domain causal influence. A similar procedure is carried out to evaluate causality from spike trains $N_1(t)$ to LFP $x(t)$ after reversing $x(t)$ and $N_1(t)$ in equation (2-10).

3. Simulation results

Multiple realizations of simulated data were generated using two-node and three-node network models where network connectivity is known. By correctly recovering the network connectivity by applying the nonparametric GC method to the mixed signals generated by the models we provide the validation for the method. It is worth noting that there are few methods which can assess the causal interactions between mixed signals that can be applied to diverse application domains.

Example 1: Two-node model

Consider the following three simple two-node models. From the first model, Equation (1), two nodes were coupled in such a way that the output of the continuous-valued time series $x(t)$ was fed into the point process data $N(t)$.

$$x(t) = 0.8x(t-1) - 0.7x(t-2) + \varepsilon_t$$
$$N(t) = \begin{cases} 1, & if\ Y(t) + 1_{\{U<0.15\}}x(t-1) > 0 \\ 0, & otherwise \end{cases} \tag{2}$$

The second model, Equation (3), also describes unidirectional coupling, but the directional causality is from point process data $N(t)$ to continuous-valued time series $x(t)$.

$$x(t) = 0.7x(t-1) - 0.5x(t-2) - 0.7N(t-1) + \varepsilon_t$$
$$N(t) = \begin{cases} 1, & if\ Y(t) > 0 \\ 0, & otherwise \end{cases} \tag{3}$$

The last model, Equation (4) illustrates a bidirectionally coupled model.

$$x(t) = 0.9x(t-1) - 0.7x(t-2) + 0.4N(t-1) + \varepsilon_t$$
$$N(t) = \begin{cases} 1, & if\ Y(t) + 1_{\{U<0.15\}}x(t-1) > 0, \\ 0, & otherwise \end{cases} \tag{4}$$

In the above models ε_t is Gaussian white noise process with zero mean and variance $\sigma^2 = 0.3$, $Y(t)$ is a Poisson process with $\lambda = 0.1$, and U is a uniformly distributed random variable between 0 and 1. We define $1_{\{U<0.15\}}$ to be 1 if an arbitrary uniform variable is less than 0.15 and 0 otherwise. For each model, we generated a data set of 1000 realizations of 1000 time points each. The sampling interval is 1ms and bin size is 1ms. The schematic diagrams of the above models are shown in the left side of Fig. S1 and the corresponding GC results are illustrated in the right side of Fig. S1. It can be seen that the nonparametric GC clearly recovers the pattern of connectivity of the models.

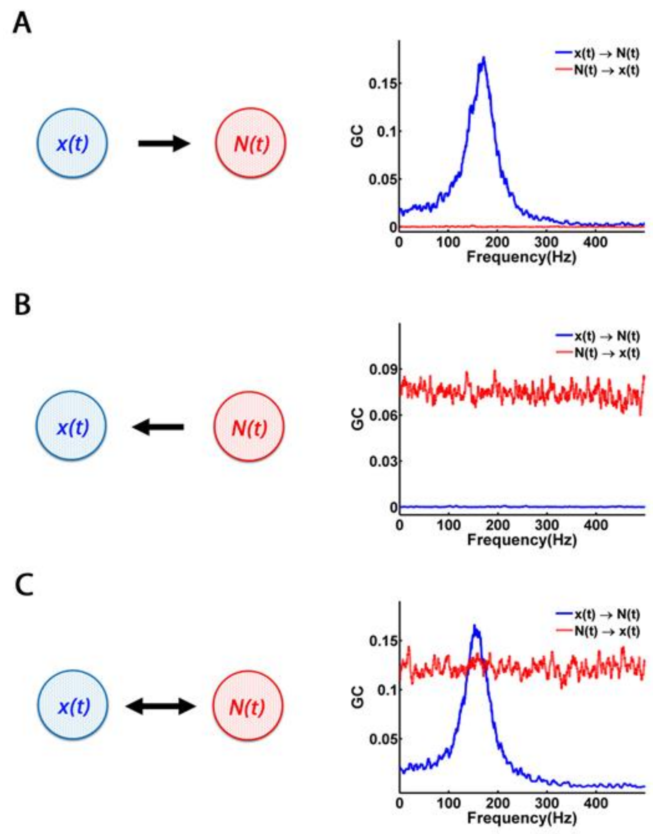

Figure S1. Simulation results for two coupled processes. Three distinct patterns of connectivity between continuous time series x(t) and point process data N(t). (A) Unidirectional coupling from continuous time series to point process, (B) unidirectional coupling from point process to continuous time series, and (C) bidirectional coupling.

Example 2: Three-node model

In this example, we consider two models each consisting of two continuous-valued time series and one point process. The first model, Equation (5), simulates the case in which influences from continuous-valued time series $x(t)$ to point process data $N(t)$ is indirect and completely mediated by $z(t)$ which is continuous time series:

$$x(t) = 0.8x(t-1) - 0.7x(t-2) + \varepsilon_t$$
$$z(t) = 0.7z(t-1) - 0.4z(t-2) - 0.7x(t-1) + \omega_t \quad (5)$$
$$N(t) = \begin{cases} 1, & if\ Y(t) + 1_{\{U<0.15\}}z(t-1) > 0. \\ 0, & otherwise \end{cases}$$

The second model, Equation (6), generates bidirectional interaction between continuous time series $x(t)$ and point process $N(t)$ with a common source $z(t)$.

$$x(t) = 0.9x(t-1) - 0.6x(t-2) + 0.4N(t-1) + 0.5z(t-1) + \varepsilon_t$$
$$z(t) = 0.8z(t-1) - 0.4z(t-2) + \omega_t \quad (6)$$
$$N(t) = \begin{cases} 1, & if\ Y(t) + 1_{\{U<0.15\}}x(t-1) + 1_{\{U<0.15\}}z(t-1) > 0 \\ 0, & otherwise \end{cases}.$$

For both models, ε_t and ω_t are two independent Gaussian white noise processes with zero means and variances of σ_1^2 = 0.2 and σ_2^2 = 0.3, respectively. Poisson process $Y(t)$ has a parameter $\lambda =$

0.1 and U is a uniformly distributed random variable between 0 and 1. We set $1_{\{U<0.15\}}$ to 1 if a uniformly distributed variable is less than 0.15 and 0 otherwise. Each model was simulated to generate a data set of 1000 realizations of 1000 time points each like in Example 1. Pairwise nonparametric GC was performed on the simulated data set of each model and generated expected results as shown in Fig. S2. Given that the experimental recordings consisted of two recording sites, we considered here mainly the pairwise GC analysis. Further dissection of the network connectivity can be achieved by applying conditional GC (results not shown) which allows the identification of direct versus indirect causal interactions as well as delineating the influence of common input (Chen et al., 2006).

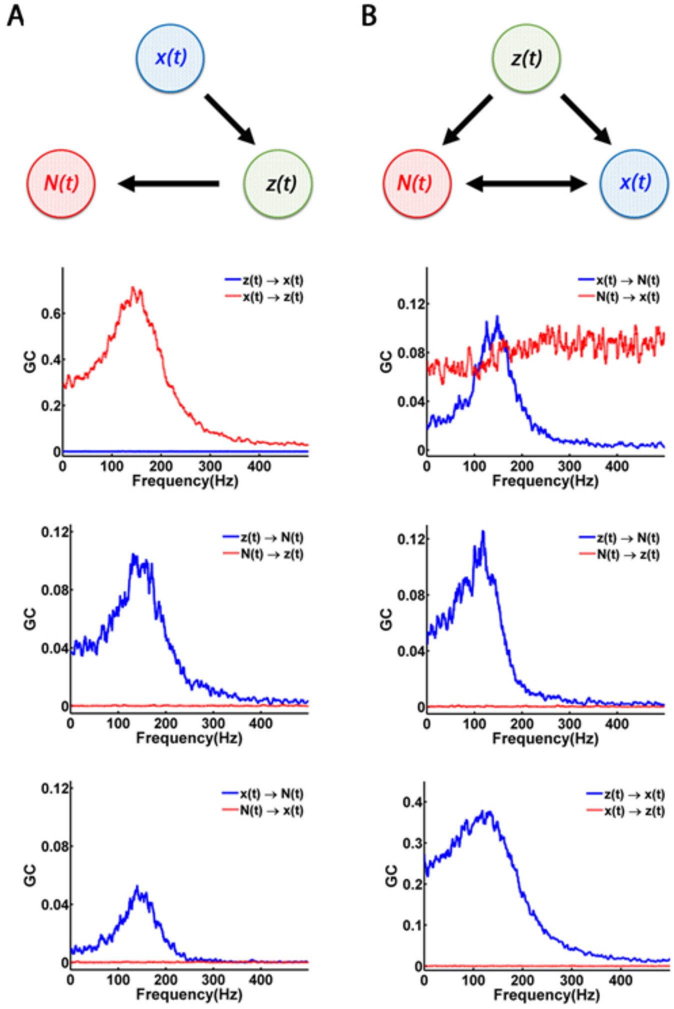

Figure S2. Simulation results for three coupled processes. Two distinct patterns of connectivity among two continuous time series x(t), z(t) and point process N(t). (A) Continuous time series x(t) has an indirect influence on N(t) via z(t) (B) Continuous time series z(t) has a direct influence on x(t) and N(t).

4. References

Dhamala, M., Rangarajan, G., & Ding, M. (2008a). Analyzing information flow in brain networks with non-parametric Granger causality. Neuroimage, 41, 354-362.

Dhamala, M., Rangarajan, G., & Ding, M. (2008b). Estimating Granger causality from fourier and wavelet transforms of time series data. Phys Rev Lett, 100, 018701.

Ding, M., Bressler, S. L., Yang, W., & Liang, H. (2000). Short-window spectral analysis of cortical event-related potentials by adaptive multivariate autoregressive modeling: data preprocessing, model validation, and variability assessment. Biol Cybern, 83, 35-45.

Ding, M., Chen, Y., Bressler, S. L. (2006). Granger causality: Basic theory and applications to neuroscience. In: Handbook of Time Series Analysis (Schelter B, Winterhalder M, Timmer J, eds), pp 437-460. Weinheim: Wiley-VCH.

Wilson, G.T. (1972). The factorization of matricial spectral densities. SIAM Journal on Applied Mathematics, 23, 420-426.
